# ASSOCIATIONS BETWEEN CHRONIC PHYSICAL CONDITIONS, MEDICATIONS AND MENTAL HEALTH IN AN AGING COHORT

**DOI:** 10.1093/geroni/igz038.2309

**Published:** 2019-11-08

**Authors:** Lucy Stirland, Lucy E Stirland, Tom C Russ, Craig W Ritchie, Graciela Muniz Terrera

**Affiliations:** 1 University of Edinburgh, Centre for Clinical Brain Sciences, Edinburgh, United Kingdom; 2 Centre forClinical Brain Sciences, University of Edinburgh, Edinburgh, Scotland, United Kingdom; 3 Alzheimer Scotland Dementia Research Centre, Edinburgh, Scotland, United Kingdom; 4 Centre for Dementia Prevention, Edinburgh, Scotland, United Kingdom; 5 Centre for Clinical Brain Sciences ,University of Edinburgh, Edinburgh, Scotland, United Kingdom

## Abstract

Older people increasingly live with multiple chronic conditions and medications. We explored their interactions with mental health in the PREVENT Dementia study participants. Using logistic and linear regression, we investigated the association between increasing self-reported chronic physical conditions and current medications with self-reported depression and anxiety disorder, and scores on the Center for Epidemiologic Studies Depression (CES-D) scale and Spielberger State-Trait Anxiety Inventory (STAI) state subtest. Among 210 participants, each additional condition was associated with increased odds of depression (adjusted OR, 95% CI: 1.41, 1.11-1.80; P=0.005) and anxiety (1.71, 1.35-2.21; P<0.001). Each additional medication was associated with depression (1.36, 1.07-1.73; P=0.010) but not anxiety. For each additional condition, CES-D scores increased by 0.62 (0.04-1.20, P=0.035) and for each medication, by 0.66 (0.12-1.21, P=0.017). There was no significant association between conditions or medications and STAI scores. These findings provide crucial information on the future brain health of these individuals.

